# Mitochondrial DNA Efflux Maintained in Gingival Fibroblasts of Patients with Periodontitis through ROS/mPTP Pathway

**DOI:** 10.1155/2022/1000213

**Published:** 2022-06-08

**Authors:** Jia Liu, Yanfeng Wang, Qiao Shi, Xiaoxuan Wang, Peihui Zou, Ming Zheng, Qingxian Luan

**Affiliations:** ^1^Department of Periodontology, Peking University School and Hospital of Stomatology, Beijing, China; ^2^National Center of Stomatology & National Clinical Research Center for Oral Diseases & National Engineering Research Center of Oral Biomaterials and Digital Medical Devices, China; ^3^Department of Physiology and Pathophysiology, Peking University Health Science Center, Beijing, China

## Abstract

Mitochondria have their own mitochondrial DNA (mtDNA). Aberrant mtDNA is associated with inflammatory diseases. mtDNA is believed to induce inflammation via the abnormal mtDNA release. Periodontitis is an infectious, oral inflammatory disease. Human gingival fibroblasts (HGFs) from patients with chronic periodontitis (CP) have shown to generate higher reactive oxygen species (ROS) that cause oxidative stress and have decreased mtDNA copy number. Firstly, cell-free mtDNA was identified in plasma from CP mice through qRT-PCR. Next, we investigated whether mtDNA efflux was maintained in primary cultures of HGFs from CP patients and the possible underlying mechanisms using adenovirus-mediated transduction live cell imaging and qRT-PCR analysis. Here, we reported that mtDNA was increased in plasma from the CP mice. Additionally, we confirmed that CP HGFs had significant mtDNA efflux from mitochondria compared with healthy HGFs. Furthermore, lipopolysaccharide (LPS) from *Porphyromonas gingivalis* can also cause mtDNA release in healthy HGFs. Mechanistically, LPS upregulated ROS levels and mitochondrial permeability transition pore (mPTP) opening by inhibition of pyruvate dehydrogenase kinase (PDK)2 expression, resulting in mtDNA release. Importantly, mtDNA efflux was even persistent in HGFs after LPS was removed and cells were passaged to the next three generations, indicating that mtDNA abnormalities were retained in HGFs in vitro, similar to the primary hosts. Taken together, our results elucidate that mtDNA efflux was maintained in HGFs from periodontitis patients through abnormal ROS/mPTP activity. Therefore, our work indicates that persistent mtDNA efflux may be a possible diagnostic and therapeutic target for patients with periodontitis.

## 1. Introduction

Periodontal inflammation is known to affect 20%-50% of the global population, and it often interacts with other inflammatory diseases such as heart disease and diabetes [1–3]. Periodontitis is associated with lipopolysaccharide (LPS) from the cell walls of gram-negative bacteria-mediated inflammatory responses and represents the most common cause of teeth loss [1, 4, 5]. Increasing evidences suggest that mitochondrial dysfunction appears to result in periodontitis during LPS stimulus [6–8]. As such, abnormal mitochondria are considered to be one of the major contributors to the periodontitis development. Several mitochondrial components, including mitochondrial DNA (mtDNA), have also been implicated in inflammatory responses [9].

mtDNA exists in mitochondrial matrix and is in intimate contact with the electron transport chain, one of the principal sources of reactive oxygen species (ROS). Therefore, mtDNA is particularly susceptible to oxidation, which can cause mutations and damages, leading to the pathogenesis of inflammation [10]. ROS production and ROS produced in mitochondria (mtROS) are indeed significantly enhanced in human gingival fibroblasts (HGFs) after LPS stimulation or from periodontitis hosts [11]. These results indicate that oxidative stress is induced during periodontitis [12]. According to current studies linking oxidative stress to decreased mtDNA copy number [13, 14], it is becoming clear that mtDNA disruption may be associated with chronic inflammation [15]. Consistent with this assumption, previous research has also demonstrated that mtDNA deletion is present in the gingival tissues of patients with periodontitis [16]. Moreover, a decrease in mtDNA in periodontitis rats suggested that aberrant mtDNA might contribute to aggravated periodontitis [7]. Considering our recent observation that HGFs and gingival tissues of patients with periodontitis in vitro had decreased mtDNA levels and decreased mitochondrial matrix protein expression, especially in pyruvate dehydrogenase kinase 2 (PDK2) when compared with those from healthy subjects [8], suggesting that mtDNA and mitochondria disruption in peripheral HGFs might replicate the mitochondrial dysfunction observed in vivo during periodontitis development. Therefore, we hypothesized that abnormal mtDNA might be maintained in HGFs in vitro connecting the disease in vivo with a certain mechanism. Answering these problems will improve our understanding of the periodontitis etiology, and it might lead to new treatment options.

Superresolution imaging demonstrated that mtDNA constitutes one copy of mtDNA and a number of different proteins, presenting densely compacted nucleoids [17]. Mitochondrial transcription factor A (TFAM) is the most notable mtDNA nucleoid protein that can be assumed to specifically recognize mtDNA [15]. mtDNA can escape from mitochondria and release into the cytoplasm under various pathological situations [18, 19]. Multiple major factors have been attributed to driving mtDNA release from damaged mitochondria, including the opening of mitochondrial permeability transition pore (mPTP), mitochondrial stress, and calcium overload [20–22]. Nonetheless, the biological mechanisms provide limited information on this process during periodontitis conditions. Our recent study demonstrated that mitochondria in HGFs from periodontitis patients appeared to retain many of the damaged features, as observed in donors [8]. In addition, previous studies have suggested that the primary host has profound influence on the cells in vitro, such as higher oxidative stress in HGFs of periodontitis patients than that of healthy individuals [8, 23]. However, differences of mtDNA release in HGFs from chronic periodontitis (CP) patients and healthy HGFs have not been tested and if this abnormal intracellular mtDNA activity would last need to be further elucidated. Thus, therapies targeting mtDNA may become a potential approach to patients suffered from severe recurrent periodontitis.

The mPTP spans the mitochondrial inner membrane, and its formation is associated with various cellular stresses [24, 25]. Interestingly, the opening of mPTP has been detected in the metabolic stress observed in inflammatory diseases [26]. More recently, using an in vitro and in vivo approach, studies have shown that genetic removal of one of the mPTP component proteins ameliorated mtDNA release into the cytoplasm during the neuroinflammatory response [20]. Pharmacological inhibition of mPTP by cyclosporin A (CsA) has also been shown to be effective in preventing mtDNA leakage into the cytoplasm [20]. Despite these results in previous studies, the notion that the opening of mPTP may directly drive mtDNA efflux remains controversial and is still unclear in periodontitis. It has been reported that ROS contributes directly to mPTP opening during ischemia-reperfusion [27]. As a result of cellular ROS and mtROS outburst, mPTP opening can be activated. Nevertheless, its association with mPTP involved in mtDNA efflux in periodontitis is scarcely understood.

In this study, we discovered the differences in the mtDNA efflux process, ROS levels, and mPTP opening between primary HGFs, isolated from patients with CP and age-matched periodontally healthy patients. This elevated mtDNA efflux together with high ROS levels, and mPTP opening in CP HGFs could be more enhanced in response to LPS. Furthermore, this identified mtDNA efflux as an important modifier could be maintained in HGFs even withdrawing the LPS stimulation after passages. Consequently, we explored if the ROS/mPTP pathway involving in the mtDNA efflux along with the progression of periodontitis. This may be a promising target for early diagnosing periodontitis and provides preclinical evidence for therapeutic strategy to people with periodontal inflammation tolerating common anti-microorganism therapy.

## 2. Materials and Methods

### 2.1. Ethics Approval

The study was approved by the Review Board and Ethics Committee of Peking University Health Science Center (PKUS-SIRB-2013017) and conducted in agreement with the Declaration of Helsinki II. Written informed consent was obtained from all subjects before inclusion in the study. All animal work was approved by the Review Board and Ethics Committee of Peking University Health Science Center (LA2018076).

### 2.2. Animals and Experimental Groups

**Figure 1:**
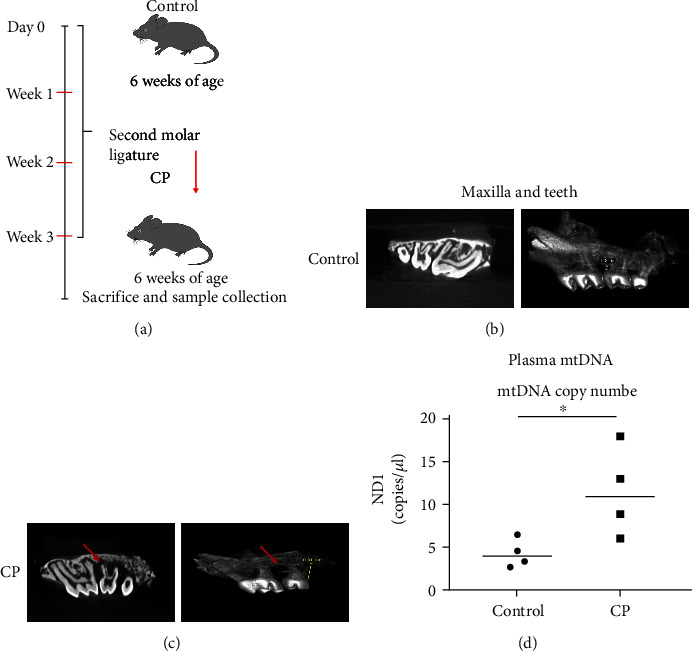
Cell-free- (cf-) mtDNA in plasma from chronic periodontitis mice and control healthy mice. (a) 5-0 silk suture was sutured for three weeks passing around the maxillary second molar in 6-week-old mice for establishing experimental chronic periodontitis (CP) mouse model. Control normal mice had no treatment. (b–c) Micro-CT showed obviously increased bone loss in CP mice after ligation for three weeks when compared to the control group. One representative image for 2-dimensional and 3-Dmode is shown. The red arrow represents bone loss areas. The yellow line indicates the distance between cement-enamel junction (CEJ) and alveolar bone crest (AEJ). (d) ND1 levels in plasma between CP and control mice (*n* = 4). ^∗^*p* < 0.05.

Specific-pathogen-free male C57BL/6 wild-type mice (6-wk-old) (Figure 1(a)) were purchased from Experimental Animal Laboratory, Peking University Health Science Center, in compliance with established polices. All mice were randomly divided into the normal control groups or CP groups of four mice each. The control group was left untreated, and the CP group had their maxillary second molar tooth ligated with a 5-0 silk ligature (Roboz Surgical Instrument Co, MD, USA) (Figure 1(a)). The ligatures remained in place in CP groups throughout the experimental period. All mice were sacrificed at three weeks postligation (Figure 1(a)). Microcomputed tomography (CT) was used for assuring the CP model was established successfully.

### 2.3. Microcomputed Tomography

In brief, after sacrificing the mice, the maxillary teeth were carefully dissected and soft tissues were removed. The sample was fixed with 4% paraformaldehyde for 24 h, and scanned using the *μ*CT50 (Scanco Medical) with a resolution of 1024 × 1024, pixel size of 15 × 15 *μ*m, and layer spacing of 15 *μ*m. The region of interest was assessed by 3D reconstructed. Bone loss was evaluated by 3D micro-CT.

### 2.4. Human Subjects

HGFs were obtained from six CP patients and six age-matched healthy donors. These participants were recruited from the Department of Periodontology, Peking University School and Hospital of Stomatology. The exclusion criteria included smoking and systemic health issues including hypertension, diabetes, and immune-related diseases within the past six months. CP was defined according to the American Academy of Periodontology and European Federation of Periodontology criteria based on staging and grading [28]. CP patients included in this study were grade B and stage III. Gingival tissues from CP were acquired through flap surgery, with PD ≥ 6 mm. Tissues in the healthy group were harvested during crown lengthening surgery, with PD < 4 mm.  Table 1 lists the detailed characteristics of the participants.

### 2.5. Primary Culture of HGFs

HGFs were prepared from the gingival tissues of six CP patients and six healthy controls during periodontal surgery. Cells were grown in Dulbecco's modified Eagle's medium supplemented with 10% fetal bovine serum (Gibco, Thermo Fisher Scientific, USA) and 1% penicillin-streptomycin. The cells were incubated at 37°C with 5% CO_2_. The medium was changed after a week. In approximately two weeks, the cells reached subconfluency and the pieces of gingival tissue were removed from the culture flask. Cells from the third to the eighth passage were used in the subsequent study.

### 2.6. Cell Treatment and Stimulation

HGFs from healthy and CP patients were stimulated with or without 5 *μ*g/mL LPS from *Porphyromonas gingivalis* (*P.g*) (ATCC33277, Standard, InvivoGen, San Diego, CA, USA) for 24 h. To investigate whether inflammatory features of donors were retained in HGFs, HGFs from healthy donors were treated with 5 *μ*g/mL of LPS for 24 h, followed by discarding the medium then passaging to the next three generations for analysis. The cells were assessed by indicated assays and compared with cells from healthy donors that were directly stimulated with the same amount of LPS for the same time.

### 2.7. Cellular ROS and Mitochondrial ROS (mtROS) Detection

2′,7′-Dichlorodihydrofluorescein diacetate (H2DCF-DA) (Sigma-Aldrich, St. Louis, MO) and MitoSOX Red (Invitrogen, Carlsbad, CA) were used to detect total ROS and mtROS, respectively, as previously described [11]. HGFs were loaded with H2DCF-DA (10 *μ*M) or MitoSOX Red (5 *μ*M) for 30 min and then observed using a microscope [11]. To inhibit ROS levels, HGFs were preincubated with 3 mM N-acetylcysteine (NAC) (Sigma Aldrich, St. Louis, MO) for 2 h. The mtROS scavenger 50 *μ*M Mito-TEMPO (Santa Cruz Biotech, Dallas, TX) were pretreated for 2 h.

### 2.8. Western Blotting

Proteins were extracted from HGFs using ice-cold radioimmunoprecipitation (RIPA) lysis buffer (Solarbio). After being quantified by BCA (Thermo Fisher Scientific), the protein samples were mixed with loading buffer (Solarbio), separated by electrophoresis on SDS-PAGE. The proteins in the gel were transferred on a polyvinylidene fluoride (PVDF) membrane (Beyotime). The membranes were blocked with 5% skimmed milk (Solarbio) and incubated overnight at 4°C with primary antibody. The membranes were washed with Tris-buffered saline and incubated with secondary antibody for 90 min at room temperature. The PVDF membranes were subjected to chemiluminescence detection using an ECL Western Blotting Detection Kit (Solarbio).

### 2.9. DNA Isolation and mtDNA Quantification by Quantitative Real-Time Polymerase Chain Reaction (qRT-PCR)

Genomic DNA from HGFs was extracted using the Universal Genomic DNA Kit (ZOMAN, Beijing, China), following the manufacturer's instructions. The mtDNA levels in HGFswere assessed using primers against mitochondrial genes (ND1), while nuclear 18S rRNA served as a loading control. Detailed ND1 and 18S rRNA sequences are presented in Table 2. Cytosolic mtDNA extraction was performed according to the methods established by West et al. [29]. The plasma from the mice was centrifuged at 1000 g for 5 min, and then the supernatant was centrifuged a second time at 5000 g for 10 min. The top 80% of the volume can be used for cell-free- (cf-) mtDNA quantification. DNA from cell supernatants, cf-mtDNA in plasma, and cytosol DNA (200 *μ*L) were isolated using the QIAamp DNA Mini Kit (Qiagen, Germany). ND1 levels in the samples were analyzed according to a standard curve based on ND1 plasmid (Sangon, Shanghai, China) levels.

### 2.10. Adenovirus Transduction for Mitochondria and mtDNA Detection

HGFs were transduced with adenovirus encoding the mitochondrial outer-membrane protein Tomm 20 bearing a mCherry fluorescence protein. mtDNA was detected by coexpression of TFAM, tagged with the green fluorescent protein (GFP) variant mNeonGreen. HGFs were seeded on 10 mm round confocal glass coverslips at a density of 50% and were infected with specified amounts of the Tomm 20-mCherry and TFAM-mNeonGreen adenoviruses. Forty-eight hours after transduction, the medium was changed, and the cells were processed for further analysis.

### 2.11. Live Cell Imaging Microscopy

Live cells were captured using a fluorescence microscope (TCS-STED; Leica, Wetzlar, Germany) with a 63 × oil immersion objective. For all experiments, HGFs were grown in 10 mm round glass bottom confocal wells (Cedarlane, Southern Ontario, Canada). Laser excitation was achieved at 488 nm for mNeonGreen and 561 nm for mCherry. LPS treatment was performed after sample mounting in the medium chamber, if needed. HGFs expressing Tomm 20-mCherry and TFAM-mNeonGreen were imaged serially at every 10 s for 10-15 min. Image processing and analysis were performed using ImageJ (NIH, http://rsb.info.nih.gov/ij/) and Huygens Professional software (Scientific Volume Imaging, Amsterdam, Holland).

### 2.12. Detection of mPTP Opening

HGFs were incubated with 50 mM cobalt chloride for 15 min, before treatment with 1 *μ*M Calcein Green AM (Solarbio, Beijing, China) for 30 min. Free Calcein quenching by cobalt chloride preserved mitochondrial integrity, which could be used to indicate mPTP opening. Calcein fluorescence was detected by confocal microscopy (Leica) using a 488 nm excitation wavelength. Quantification of the Calcein fluorescence intensity was conducted by analyzing 20 cells for every indicated condition using ImageJ software. To prevent mPTP opening, HGFs were preincubated with 0.5 *μ*M cyclosporine A (CsA; Sigma) for 2 h, following the manufacturer's recommendations.

### 2.13. Flow Cytometric Analysis

Cells were briefly washed with 1 × phosphate-buffered saline (PBS), resuspended in 1 × binding buffer, and centrifuged at 300 × g for 10 min. The pellets were resuspended with 1 × binding buffer at a density of 1 × 10^6^ cells/mL. Cells were replated in a flow cytometric tube at a density of 1 × 10^5^ cells/mL and processed for Annexin V-FITC staining (Solarbio, Beijing, China)for 10 min at 20-25°C. Subsequently, the cells were stained with propidium iodide (PI) for 5 min at 20-25°C and analyzed for apoptosis by flow cytometry.

### 2.14. Statistical Analysis

Data are expressed as the mean ± standard error (SE). All *p* values were determined by two-way Student's *t*-test or one-way analysis of variance (ANOVA) with a post hoc Student Knewman-Keuls test for multiple comparisons. Significant differences were accepted at *p* < 0.05. Statistical analysis was performed using GraphPad Prism software (version 9.00; GraphPad Software).

## 3. Results

### 3.1. mtDNA Release from Mitochondria during Periodontitis Development

**Figure 2:**
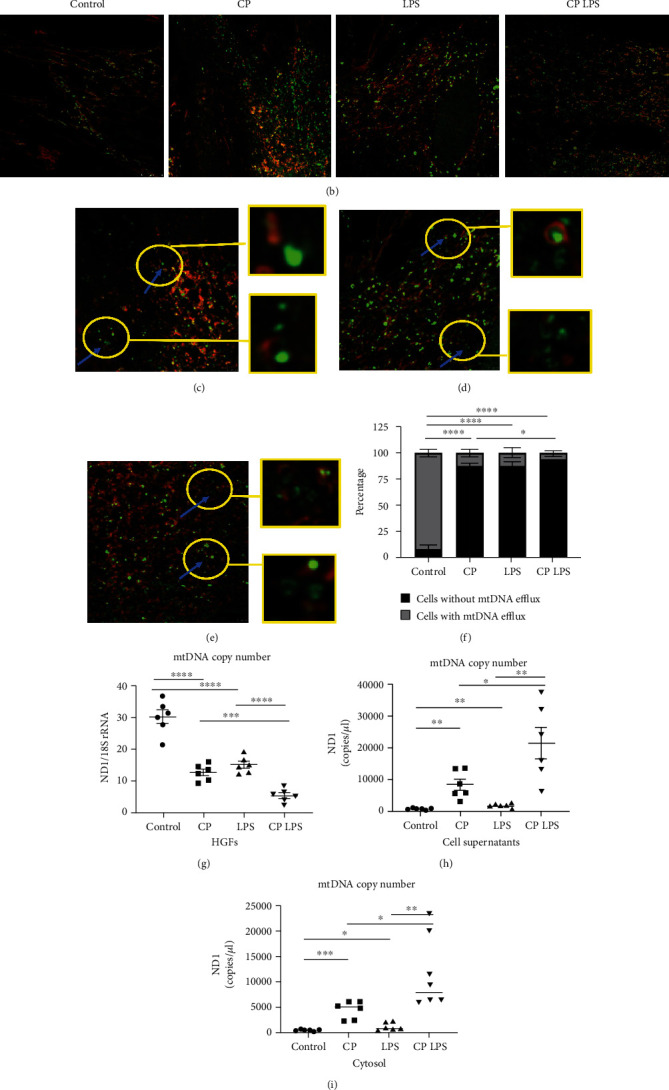
mtDNA released from mitochondria in human gingival fibroblasts from patients with chronic periodontitis. (a) Using fluorescent fusion proteins to visualize mitochondria (Tomm 20-mCherry) and mtDNA (TFAM-mNeonGreen). TM: transmembrane domain; MLS: mitochondrial localization sequence; DBD1 and DBD2: DNA binding domain-1 and DNA binding domain-2. (b) Typical illustration of the human gingival fibroblasts (HGFs) for mitochondria and mtDNA among control, CP, and with or without LPS stimulation (5 *μ*g/mL, 24 h) groups (scale bars: 5 *μ*m). (c) Still image of mtDNA efflux in HGFs from CP patients (scale bar: 2.5 *μ*m, see Movie 1). (d) Still image of mtDNA efflux in HGFs with LPS stimulation (scale bar: 2.5 *μ*m, see Movie 2). (e) Still image showing mtDNA efflux in HGFs from CP patients with LPS stimulation (scale bar: 2.5 *μ*m, see Movie 3). (f) Percentage of HGFs with, without mtDNA efflux. Data are the mean ± SE of 20 fields in each group. (g–i) mtDNA in HGFs, cell supernatants, and cell cytosol. Data are obtained by six independent experiments. ^∗^*p* < 0.05,  ^∗∗^*p* < 0.01,  ^∗∗∗^*p* < 0.001, and^∗∗∗∗^*p* < 0.0001.

Micro-CT results revealed that alveolar bone around the ligated molar was significantly reduced in CP mice compared to control mice, suggesting experimental periodontitis in the CP group established (Figures 1(b) and 1(c)). Intriguingly, mtDNA in plasma from CP mice were enriched compared to age-matched wild-type control mice (Figure 1(d)). These results indicated that mtDNA release might be involved in periodontitis development. However, mtDNA efflux in HGFs during periodontitis is still unclear. Next, we transduced primary HGFs with adenovirus encoding Tomm 20-mCherry and TFAM-mNeonGreen to show mitochondria and mtDNA, respectively (Figure 2(a)). mtDNA were detected robust release into the cytoplasm in CP HGFs (Figure 2(b)). This process was also found by real-time microscopy (Figure 2(c), Movie 1). In contrast, no mtDNA efflux was detected in healthy HGFs (Movie S1). LPS caused remarkable mtDNA release in healthy HGFs and led to more significant mtDNA release in periodontitis-affected samples (Figures 2(b)–2(d), and 2(e), Movie 2 and 3).Next, we calculated a significant increase in the percentage of HGFs with mtDNA efflux in CP HGFs as compared with that in control HGFs (Figure 2(f)). LPS treatment caused marked increase in the percentage of HGFs with mtDNA efflux compared with those without LPS treatment in healthy and CP states (Figure 2(f)). Moreover, qRT-PCR confirmed that mtDNA release into cytosol and out of cells during periodontitis (Figures 2(g)–2(i)). These results indicated that mtDNA release might be involved in periodontitis development.

### 3.2. mtDNA Efflux Maintained in HGFs during Periodontitis

**Figure 3:**
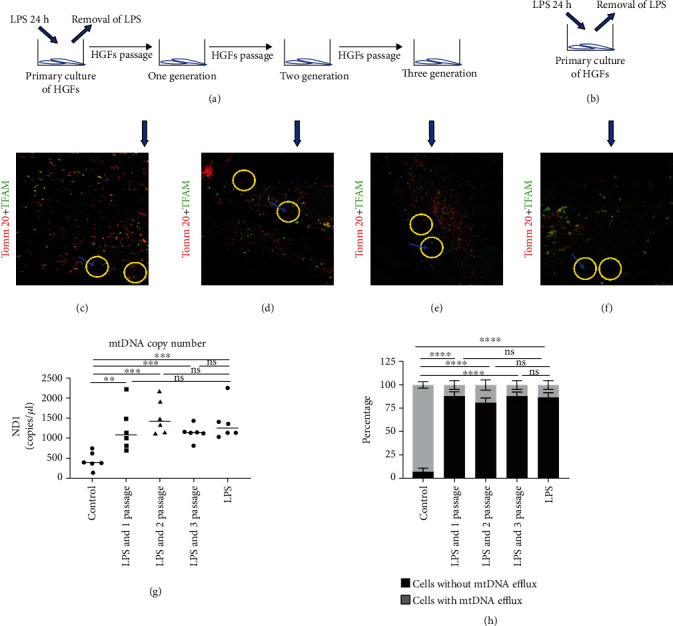
mtDNA efflux maintained inhuman gingival fibroblasts during periodontitis. (a) Human gingival fibroblasts (HGFs) were treated with lipopolysaccharide (LPS) (5 *μ*g/mL, 24 h), which was later removed from the culture medium. These LPS-treated HGFs were cultured to next three generations for analysis. (b) HGFs were cultured with LPS direct stimulation (5 *μ*g/mL, 24 h), which will be directly analyzed for mtDNA activity. (c–e) Images of HGFs in the A group for analysis after passaging three generations, respectively. (f) Images of HGFs in the B group. Scale bars: 5 *μ*m. (g) mtDNA levels in cytosol among the five groups of HGFs. *n* = 6. (h) Percentage of HGFs with, without mtDNA efflux. Data in (h) are the mean ± standard error (SE) of 20 fields per group. ns = no significant difference. ^∗∗^*p* < 0.01,  ^∗∗∗^*p* < 0.001, and^∗∗∗∗^*p* < 0.0001.

LPS is a principal factor that determines the periodontal inflammation; we decided to clarify if LPS causes mtDNA efflux maintained in HGFs. In these experiments, healthy HGFs were exposed to LPS stimulation for 24 h. Next, LPS was removed and HGFs were cultured into the next three generations for analysis (Figure 3(a)). In contrast, healthy HGFs were directly treated with LPS for 24 h (Figure 3(b)). The results showed that LPS reinforced the mtDNA efflux effect even in the next three generational HGFs (Figures 3(c)–3(e)). No significant differences were observed compared to the LPS directly treated HGFs (Figure 3(f)). Next, we examined the mtDNA levels in the cytosol using qRT-PCR analyzing these groups. LPS directly stimulated HGFs, and LPS treatment following passages of HGFs were both enriched in cytosolic mtDNA (Figure 3(g)). In addition, the percentages of HGFs with mtDNA efflux between LPS direct treatment and LPS treatment followed by HGFs passages were similar (Figure 3(h)). These results suggest that LPS treatment can enhance this mtDNA efflux phenomenon, and the facilitative mtDNA release effects can be maintained in HGFs even in the next-generations HGFs, which is consistent with those mtDNA release of CP HGFs and CP mice.

### 3.3. ROS and mtROS Is Overproduction in HGFs from CP Patients

**Figure 4:**
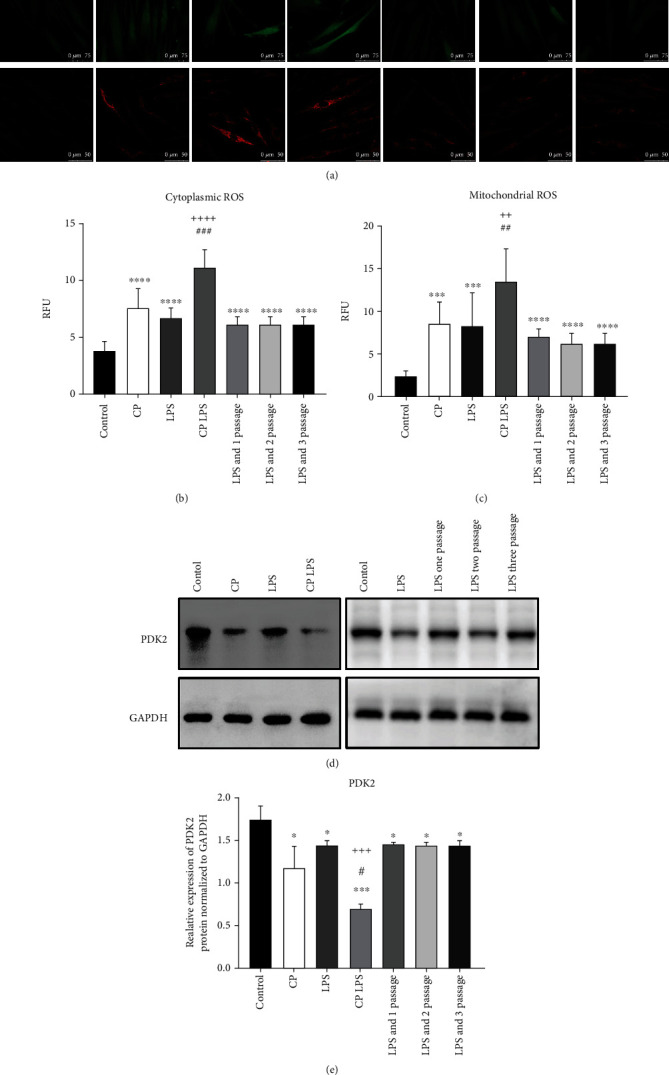
Reactive oxygen species (ROS) and mitochondrial ROS is overproduction in human gingival fibroblasts from chronic periodontitis patients. (a) Human gingival fibroblasts (HGFs) were incubated with 2′,7′-dichlorodihydrofluorescein diacetate (H2DCF-DA) (10 *μ*M, 30 minutes) to indicate the ROS levels (green) in HGFs (scale bars: 75 *μ*m). HGFs were incubated with MitoSOX Red (5 *μ*M, 30 minutes) to visualize mitochondrial ROS (mtROS) levels (red) (scale bars: 50 *μ*m). (b, c) The arbitrary fluorescence intensity of ROS and mtROS in (a) were calculated by ImageJ based on per 10 cells in each group from (a). Data represent the mean ± standard error (SE) from 10 cells from each group. (d) Western blot to evaluate the protein expression of pyruvate dehydrogenase kinase 2 (PDK2). Glyceraldehyde-3-phosphate dehydrogenase (GAPDH) as the loading control. (e) Quantification of each band intensity with respect to loading control in D. *n* = 3 (LPS: 5 *μ*g/mL). Statistically significant *p* value is indicated as follows: ^∗^*p* < 0.05,  ^∗∗∗^*p* < 0.001, and^∗∗∗∗^*p* < 0.0001 as compared with the control group; ^#^*p* < 0.05,  ^##^*p* < 0.01, and^###^*p* < 0.001 as compared with the CP group; ^++^*p* < 0.01,  ^+++^*p* < 0.001, and^++++^*p* < 0.0001 as compared with the LPS group.

To investigate in further details how mtDNA efflux effect remained during periodontitis at cellular level, we firstly analyzed the ROS and mtROS levels in HGFs from different hosts. Control healthy HGFs had the lowest ROS and mtROS levels (Figure 4(a)). HGFs from CP had significantly greater levels of ROS and mtROS (Figure 4(a)). We found that the ROS and mtROS were more activated in the presence of LPS compared to those in the absence of LPS groups (Figure 4(a)). In addition, LPS can affect ROS and mtROS even in the next three generations HGFs (Figure 4(a)). Furthermore, the levels of these fluorescent signals reflecting ROS and mtROS levels in Figure 4(a) were calculated by ImageJ (Figures 4(b) and 4(c)). Western blot analysis showed PDK2 exhibited decreased expression in CP HGFs (Figures 4(d) and 4(e)). Meanwhile, the expression levels of PDK2 were also reduced after LPS stimulation and showed low levels even in the next three-generation HGFs (Figures 4(d) and 4(e)). In summary, CP HGFs are primed for ROS activation, and LPS can persistently upregulate the ROS levels in HGFs by suppressing the PDK2 expression. Its regulation may contribute to this mtDNA efflux process.

### 3.4. mPTP Opening in HGFs from CP Patients via ROS Activation

**Figure 5:**
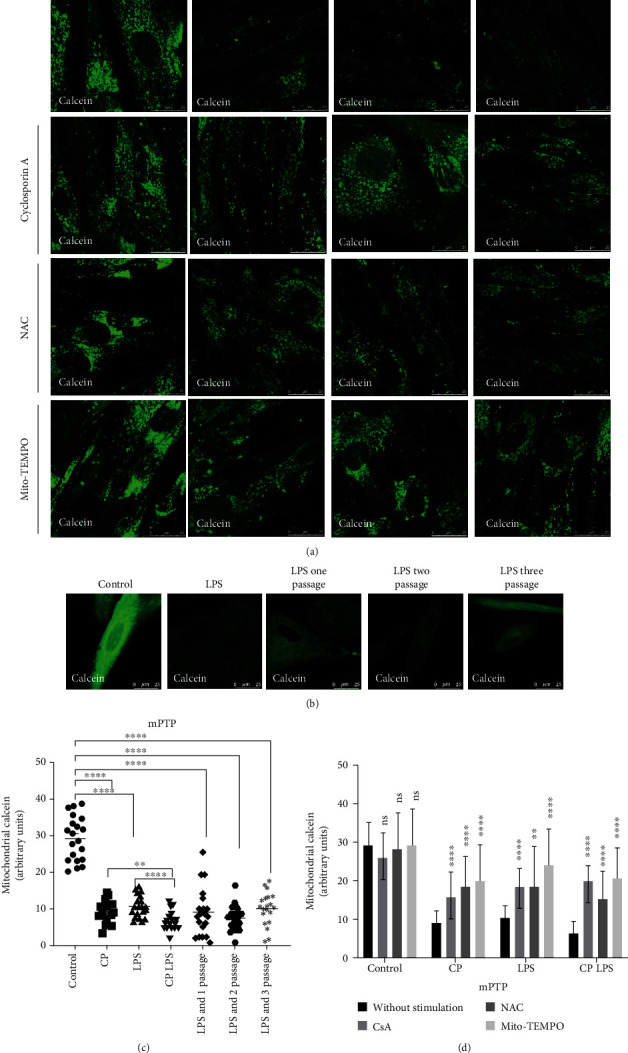
Human gingival fibroblasts (HGFs) from chronic periodontitis presented active mitochondrial permeability transition pore opening via reactive oxygen species. (a) Human gingival fibroblasts (HGFs) were loaded with cobalt chloride (50 mM, 15 minutes) and Calcein AM (green) to determine the opening of the mitochondrial permeability transition pore (mPTP) in HGFs in the presence or absence of lipopolysaccharide (LPS) treatment (5 *μ*g/mL, 24 h). The opening of mPTP in HGFs was measured after cyclosporin A (CsA) (0.5 *μ*M, 2 h) or N-acetylcysteine (NAC) (3 mM, 2 h)or Mito-TEMPO (50 *μ*M, 2 h) treatment (scale bars: 25 *μ*m). (b) Images for mPTP opening in the control, LPS-treated, and LPS-treated group after passaging three generations (scale bars: 25 *μ*m). (c) Quantification of the observed Calcein green signal in HGFs from (a, b). Mean ± SE are indicated (*n* = 20 cells). The CP group was observed a lower signal; LPS was also observeda lower signal compared with control HGFs. LPS can aggravate this lower signal in the control and CP groups, and this phenomenon can be retained in HGFs after LPS was removed and passaged to next three generations as compared with the control group. (d) The intensity of the indicated Calcein green signal was detected per 20 cells from the control, CP, LPS, and CP LPS groups with CsA, NAC, and Mito-TEMPO treatment compared to HGFs without any treatment, respectively. CsA, NAC, and Mito-TEMPO all downregulated the signal in the LPS, CP, and CP LPS groups, while they fail to induce this phenomenon in control HGFs. *p* values were determined by 1-way analysis of variance followed by post hoc tests. ^∗∗^*p* < 0.01 and^∗∗∗∗^*p* < 0.0001; ns: not significant.

mPTP opening in HGFs was indicative using Calcein AM fluorescence (Figure 5(a)). Control HGFs showed strong green fluorescence (Figure 5(a)), suggesting that mPTP remained in a closed state under normal condition [30]. However, the fluorescence was hardly detected in CP HGFs (Figure 5(a)). LPS further resulted in a much more decrease in fluorescence in the control and CP groups (Figure 5(a)). Decreased level of fluorescence signal was also detected in the LPS treated following passaging three generational HGFs (Figure 5(b)). A significant increase in fluorescence was observed in HGFs in the presence of CsA when compared with that in the absence of CsA (Figures 5(a)–5(d)). It was shown that inhibition of ROS and mtROS activation contributes to suppression of mPTP opening (Figures 5(a)–5(d)). Collectively, these data show that CP HGFs display mPTP opening and that mPTP opening in the LPS-treated HGFs was maintained within the HGFs even in the later three generations. Additionally, this observed mPTP opening is dependent on ROS activation.

### 3.5. mtDNA Release in CP HGFs via ROS and mPTP Opening

**Figure 6:**
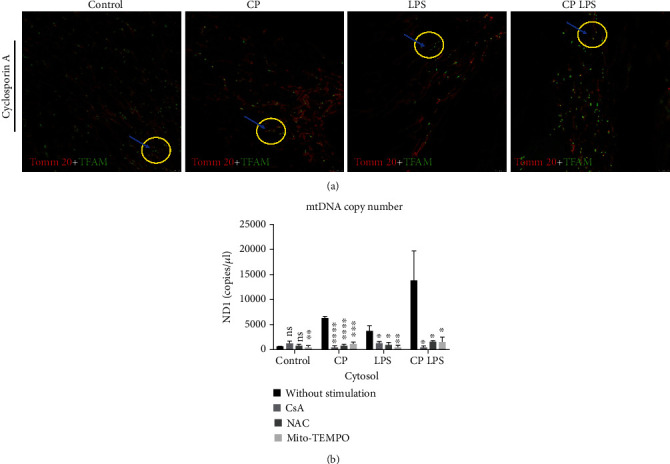
mtDNA release from mitochondria in human gingival fibroblasts of chronic periodontitis patients via reactive oxygen species-mitochondrial permeability transition pore pathways. (a) Human gingival fibroblasts (HGFs) from the control, chronic periodontitis (CP), and lipopolysaccharide (LPS) stimulation infection (5 *μ*g/mL, 24 h), and CP LPS groups were pretreated with 0.5 *μ*M cyclosporin A (CsA) for 2 h and subjected to analysis for mtDNA release. HGFs expressing Tomm 20-mCherry (red) and TFAM-mNeonGreen (green) revealed mtDNA nucleoid presented along with mitochondria. Yellow circles and blue arrows mark areas where mtDNA (green) clearly stops efflux from mitochondria (red). Scale bars: 2.5 *μ*m. See Movies 4, 5, 6, and 7). (b) Bar graphs illustrate the average mtDNA levels in cytosol among four groups of HGFs with or without CsA, N-acetylcysteine (NAC) (3 mM, 2 h), and Mito-TEMPO (50 *μ*M, 2 h) treatment. All quantified data represent the mean ± SE. *p* values were determined by 1-way analysis of variance followed by post hoc tests. Graphs represent at least 3 independent experiments. ^∗^*p* < 0.05,  ^∗∗^*p* < 0.01,  ^∗∗∗^*p* < 0.001, and^∗∗∗∗^*p* < 0.0001; ns: not significant.

We performed real-time fluorescent microscopy for control, CP, LPS treatment, and CP LPSHGFs in the presence of CsA (Figure 6(a)). It was observed that mtDNA displayed mild or no efflux in the four CsA-treated groups of HGFs (Figure 6(a)). These data demonstrated that mPTP was critical for the mtDNA release under these conditions. We performed qRT-PCR to detect the cytosolic mtDNA levels by the inhibitors of mPTP, ROS, and mtROS. CsA, NAC, and Mito-TEMPO all decreased the cytosolic mtDNA levels in the CP, LPS, and CP LPS groups when compared with the three groups without any treatment, whereas the control group showed similar cytosolic mtDNA levels in the presence and absence of CsA and NAC (Figure 6(b)). We also showed that Mito-TEMPO slightly decreased cytosolic mtDNA concentration in healthy HGFs (Figure 6(b)). When we examined the difference in apoptosis of HGFs among the four groups, we found that HGFs from CP, LPS, and CP LPS showed no significant apoptosis when compared with the HGFs of control healthy donors (Fig. S1). Cumulatively, these results provide further evidence that ROS-mPTP opening causes mtDNA release in CP and LPS-treated HGFs.

## 4. Discussion

Mitochondrial dysfunction is an important component of periodontitis pathogenesis [31], as defects in mitochondrial structure and function have been shown in periodontitis in our previous work [8]. mtDNA is crucial for mitochondrial function. It is known that mtDNA has structural similarities with microbial DNA [32]. Hence, mtDNA could result in an inflammatory response when released into the cytoplasm or extracellular milieu in susceptible patients. These mtDNA characteristics confirmed the significant role of mtDNA in the pathogenesis of inflammation-related diseases in humans. In this study, we examined mtDNA efflux activity and extent using confocal microscopy and qRT-PCR analysis between primary HGFs from periodontitis patients and healthy donors. We demonstrated for the first time that mtDNA released from the mitochondria in HGFs from CP patients. LPS stimuli was found to trigger this mtDNA efflux activity and keep these properties within the HGFs for some periods.

Studies have previously identified that mtDNA is found outside the mitochondria and sometimes even outside the cells in certain circumstances [33, 34]. mtDNA release was first reported that LPS pointed to extrude mtDNA into the cytoplasm [35]. Another key evidence for mtDNA extruding into the extracellular space is that LPS induces neutrophil extracellular traps (NETs) formation, largely consisting of mtDNA [36, 37]. This mtDNA release may result in substantial tissue damage, leading to chronic inflammation. Periodontitis is a kind of chronic inflammatory disease driving the destruction of soft and hard periodontal tissues such as gingiva recession and alveolar bone loss [5], suggesting a role for mtDNA efflux in the periodontitis. Consistent with the reported mtDNA efflux in other studies, we identified a significant increased mtDNA levels in the plasma from CP mice, implying an association with periodontitis and this mtDNA efflux. One study demonstrated that the mtDNA outside of mitochondria was found to be crucial for inflammation via inducing bone-destructing immunity [38]. Owing to the mtDNA accumulation in the plasma of CP mice, little is known about the mtDNA function and activity in HGFs during periodontitis. In the context of periodontitis, in vitro studies of periodontitis patients have confirmed alterations in mitochondrial structure, function, and hyperoxidative stress in HGFs and gingival tissues compared to normal individuals [8, 12], which indicates that there may be a correlation between periodontitis progression and mitochondrial dysfunction in HGFs from different hosts. Interestingly, we confirmed that aberrant mtDNA release into cytosol and supernatants of HGFs from CP patients. It is evident that LPS stimulation could also induce this phenomenon in healthy HGFs. The observed high mtDNA levels in the cytosol and supernatants of CP HGFs were more significant in the presence of LPS than in HGFs without LPS, indicating that mtDNA release was maintained in inflamed cells. It is possible that LPS, a major trigger of periodontitis, enables mtDNA to release from mitochondria in periodontitis mouse model and periodontitis patients, but the retained mtDNA efflux in HGFs from CP patients was inadequate to understand.

Our data showed that LPS increased ROS levels and mPTP opening, and it also led to this variation in next three generational HGFs. This comparison of mtDNA efflux activity between the next three generations HGFs after LPS stimuli and direct LPS treatment was similar, in line with the above findings. In addition, we demonstrated that LPS upregulated ROS generation through PDK2 inhibition even in the next three generations HGFs. It was reported that PDK2 activation has beneficial effects on ROS suppression [39]. Thus, we reasoned that LPS might mediate irreversible high ROS generation by downregulation of PDK2 expression [6, 40], leading to sustained increased mtDNA release activity even in the next-generation HGFs without LPS stimulation. As widely reported in the literature, LPS activated Toll-like receptor (TLR) was abundantly expressed in the inflammatory cells, leading to the ROS production as well as the lower PDK2 expression [8, 41]. Some reported that ROS triggered mtDNA damages and release into cytosol in cancer [42]. Another study showed that mitochondrial ROS induced inflammation dependent on disrupting mtDNA maintenance [15]. In agreement, other study detected that LPS induced accumulation of free mtDNA outside of mitochondria contributing to inflammation via TLR9 activation [43]. We provided herein the proof of mtDNA efflux arising from LPS mediating ROS activation by blocking PDK2 in HGFs. The exact reason for this phenomenon is unclear. It is crucial to note that studies have observed the transfer of entire mitochondria between cells [44]. However, whether the entire mitochondria or mtDNA is transferred is controversial [45]. Therefore, mtDNA is thought to be a signal molecule that spreads inflammatory signals across a population of cells. This suggests that inflammation could spread between cells via the detection of mtDNA [9]. Based on these results, we propose that LPS modulates mtDNA efflux remained in HGFs closely linked to sustained ROS overproduction.

Some studies have concluded mtDNA release in the context of cell apoptosis [46], while other studies have indicated that mPTP opening leads to increased mtDNA release [46]. Given that cell apoptosis was similar among our divided groups, the role of mPTP opening in mtDNA release in HGFs was focused. Based on the inhibitory effect of CsA on mPTP opening, CsA successfully restored mtDNA efflux from mitochondria and reduced mtDNA levels in cytosol within inflamed HGFs. These results highlight that mPTP opening potentially modulates mtDNA release in HGFs with periodontitis. One of the possible physiological mechanisms mediating increased mPTP opening could be linked to ROS increase in inflammatory HGFs. Exceeding levels of ROS can trigger mPTP opening via mitochondrial ATP-sensitive potassium channels, and voltage-dependent anion channel-1 oligomerization, suggesting that ROS works as an important molecular leading to downstream mPTP opening and eventually disruption of cellular functions [47, 48]. Of note, earlier, Bullon's work together with our recent work demonstrated that HGFs and gingival tissues from CP patients were observed impaired mitochondria and higher oxidative stress [8, 12, 49]. As a result of cellular ROS and mtROS outburst, mPTP opening can be activated. According to our data, we confirmed positive relationship between ROS overproduction and mPTP opening in inflammatory HGFs. In addition, ROS and mPTP both played a critical role in mtDNA release during periodontitis. Our results highlight that ROS could be one possible explanation for mPTP opening, contributing to mtDNA release in HGFs during periodontitis.

## 5. Conclusion

In summary, mtDNA efflux maintained in primary HGFs could reflect mitochondrial dysfunction detected in periodontitis. This work provides initial preclinical evidence for a new candidate biomarker for mtDNA efflux in HGFs predicting periodontitis. In addition to this focused investigation of mtDNA efflux in HGFs during inflammation, our results also indicate that ROS/mPTP pathway could be the principal mediator of mtDNA efflux in inflamed HGFs. Further investigation is needed to determine how mtDNA release causes periodontitis, which may reveal new therapeutic strategies for the treatment of patients with periodontitis.

## Figures and Tables

**Table 1:** Clinical characteristics at surgery site of patients included in this study.

Abbreviation	Number of patients	Range of age	Percent women	BI PD (mm)	CAL (mm)
Con	6	27-40	50	1-2	0-0.5
CP	6	33-45	66.7	6-10	4-7

**Table 2:** List of primers for real-time PCR studies.

Gene	Primer	Sequence
ND 1	Forward primer	5′-CACACTAGCAGAGACCAACCGAAC-3′
	Reverse primer	5′-CGGCTATGAAGAATAGGGCGAAGG-3′
18S rRNA	Forward primer	5′-GACTCAACACGGGAAACCTCACC-3′
	Reverse primer	5′-ACCAGACAAATCGCTCCACCAAC-3′

## Data Availability

The datasets generated and analyzed during the current study are available from the corresponding authors on reasonable request.

## Supplementary Materials

Supplementary 1 Fig. S1: measurement of cell apoptosis by Annexin V binding and propidium iodide (PI) uptake. Human gingival fibroblasts (HGFs) were from control donors (A) or chronic periodontitis (CP) patients (B). Control cells were treated with lipopolysaccharide (LPS) (5 *μ*g/mL, 24 h) (C), and CP cells were treated with LPS (5 *μ*g/mL, 24 h) (D). These four groups of HGFs were harvested for cell apoptosis analysis by flow cytometry 24 h after LPS treatment or without LPS treatment.

Supplementary 2 Movie. S1: mtDNA does not release from mitochondria in human gingival fibroblasts from healthy control donors in real time movie. Human gingival fibroblasts (HGFs) from control hosts were transduced with vector encoding the mitochondrial outer-membrane protein Tomm 20 bearing a mCherry fluorescence to illustrate mitochondria (red). Meanwhile, HGFs were also transduced with mitochondrial transcription factor A (TFAM) tagged with the green fluorescent protein (GFP) variant mNeonGreen to detect mtDNA (green) (scale bar: 7.5 *μ*m).
