# Supplementary figures and images for: Mitochondrial DNA Efflux Maintained in Gingival Fibroblasts of Patients with Periodontitis through ROS/mPTP Pathway
Source: Oxid Med Cell Longev. 2022 Jun 8;2022:1000213. doi: 10.1155/2022/1000213 (PMC9201712; doi:10.1155/2022/1000213)

Figure S1

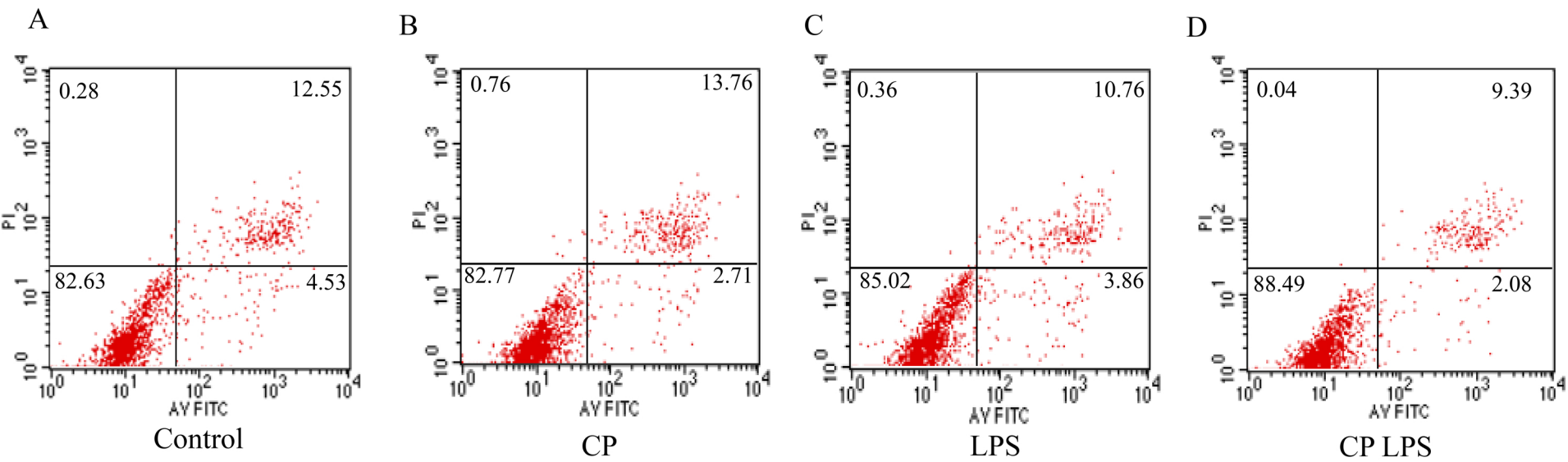

Supplement: Supplementary 1 — Fig. S1: measurement of cell apoptosis by Annexin V binding and propidium iodide (PI) uptake. Human gingival fibroblasts (HGFs) were from control donors (A) or chronic periodontitis (CP) patients (B). Control cells were treated with lipopolysaccharide (LPS) (5 μg/mL, 24 h) (C), and CP cells were treated with LPS (5 μg/mL, 24 h) (D). These four groups of HGFs were harvested for cell apoptosis analysis by flow cytometry 24 h after LPS treatment or without LPS treatment. [file 1000213.f1.pdf]
