# Supplementary material for: Mitochondrial DNA Efflux Maintained in Gingival Fibroblasts of Patients with Periodontitis through ROS/mPTP Pathway
Source: Oxid Med Cell Longev. 2022 Jun 8;2022:1000213. doi: 10.1155/2022/1000213 (PMC9201712; doi:10.1155/2022/1000213)
Supplement: Supplementary 2 — Movie. S1: mtDNA does not release from mitochondria in human gingival fibroblasts from healthy control donors in real time movie. Human gingival fibroblasts (HGFs) from control hosts were transduced with vector encoding the mitochondrial outer-membrane protein Tomm 20 bearing a mCherry fluorescence to illustrate mitochondria (red). Meanwhile, HGFs were also transduced with mitochondrial transcription factor A (TFAM) tagged with the green fluorescent protein (GFP) variant mNeonGreen to detect mtDNA (green) (scale bar: 7.5 μm). [file 1000213.f2.pdf]

Supplementary files

Movie Drive link-https://pan.baidu.com/s/1VnoTumgu3AS0t0d59_4ieA **.**

Password:dsun
